# Targeted long-read sequencing analysis and antifungal susceptibility profiles of *Sporothrix schenckii* isolates from Thailand

**DOI:** 10.1371/journal.pntd.0013253

**Published:** 2025-06-30

**Authors:** Nattapong Langsiri, Wijit Banlunara, Oranong Klaychontee, Navaporn Worasilchai, Regielly Cognialli, Flavio de Queiroz-Telles, Bram Spruijtenburg, Theun de Groot, Eelco F.J. Meijer, Ariya Chindamporn

**Affiliations:** 1 Department of Microbiology, Faculty of Medicine, Chulalongkorn University, Bangkok, Thailand; 2 Department of Veterinary Pathology, Chulalongkorn University, Patumwan, Bangkok Thailand; 3 Department of Transfusion Medicine and Clinical Microbiology, Faculty of Allied Health Science, Chulalongkorn University, Bangkok, Thailand; 4 Center of Excellence of Medical Mycology Diagnosis, Chulalongkorn University, Bangkok Thailand; 5 Mycology Unit, Hospital de Clínicas, Federal University of Paraná, Curitiba, Brazil; 6 Department of Basic Pathology, Graduate Program in Microbiology, Parasitology and Pathology, Biological Sciences, Federal University of Paraná, Curitiba, Brazil; 7 Department of Public Health, Clinical Hospital, Federal University of Paraná, Curitiba, Brazil; 8 Radboudumc-CWZ Center of Expertise for Mycology, Nijmegen, The Netherlands; 9 Department of Medical Microbiology and Immunology, Canisius-Wilhelmina Hospital (CWZ)/Dicoon, Nijmegen, The Netherlands; 10 Center of Excellence in Antimicrobial Resistance and Stewardship, Chulalongkorn University, Bangkok, Thailand; Albert Einstein College of Medicine, UNITED STATES OF AMERICA

## Abstract

*Sporothrix* spp. are dimorphic fungi capable of undergoing morphological changes in response to temperature variations. The genus *Sporothrix* includes the species *S. schenckii sensu stricto*, *S. brasiliensis*, *S. globosa*, and *S. luriei* that cause sporotrichosis, which can range from local skin infections to systemic infections in immunocompromised individuals. As these species are morphologically similar, molecular techniques that utilize barcoding genes are required for accurate identification. While the Internal Transcribed Spacer (ITS) region is the universal fungal barcode, the calmodulin gene offers higher resolution for phylogenetic classification of *Sporothrix* using Sanger sequencing. This study evaluated the ability of long-read nanopore sequencing of calmodulin and ITS to identify species level and allow phylogenetic analysis of *Sporothrix* strains isolated from humans and felines in Thailand. We found that the calmodulin sequencing with Oxford Nanopore Technology (ONT) consistently classified all isolates as *S. schenckii sensu stricto*, whereas the ITS region showed a lower discriminatory power, complicating species identification in some isolates. The phylogenetic analysis of the calmodulin region indicated that all isolates localized in a specific *S*. *schenckii sensu stricto* subclade together with other isolates from Southeast Asia, while the three residual *S*. *schenckii* subclades were associated with other geographical locations. Antifungal susceptibility testing on all *Sporothrix* strains demonstrated elevated *in vitro* minimum inhibitory concentrations (MICs) to itraconazole for 8 out of 26 isolates. Altogether, this study demonstrated that ONT sequencing of calmodulin allows accurate species identification and phylogenetic analysis of *S*. *schenckii sensu stricto* isolates from Thailand, of which some also demonstrated elevated MIC values for itraconazole.

## Author summary

This study evaluated the use of Oxford Nanopore Technology (ONT) long-read sequencing to identify and analyze species of *Sporothrix* spp., a group of dimorphic fungi that cause sporotrichosis. The research focused on the calmodulin gene as a more reliable tool for species identification and phylogenetic analysis compared to the commonly used Internal Transcribed Spacer (ITS) region. By sequencing isolates from humans and felines in Thailand, the authors found that ONT sequencing of the calmodulin gene effectively classified the isolates as *S. schenckii sensu stricto*, offering better resolution than ITS sequencing, which struggled with species discrimination. Phylogenetic analysis revealed that the isolates clustered within a specific subclade of *S. schenckii sensu stricto*, with a distinct geographic association to Southeast Asia. Additionally, the study highlighted the presence of elevated minimum inhibitory concentrations (MICs) to itraconazole in several isolates. Altogether, these findings underscore the utility of ONT calmodulin sequencing for accurate identification and phylogenetic studies of *Sporothrix*.

##  Introduction

*Sporothrix* spp. are dimorphic fungi belonging to the order Ophiostomatales, family Ophiostomataceae. These species can change from a filamentous shape to an oval budding yeast depending on temperature. The *Sporothrix* clinical clade includes the species *S. schenckii sensu stricto*, *S. brasiliensis*, *S. luriei* and *S. globosa*, and can be found in soil, plants, biological debris and on mammals, causing disease in humans and animals. Species identification within this clade is only possible with molecular techniques, as microscopy is insufficient [1,2]. Each species within the clinical *Sporothrix* clade presents significant differences in epidemiology, severity of the disease, virulence, activation of immune response, and transmission route. For example, while most *Sporothrix* species are transmitted in the mycelial phase in a sapronotic way, *S. brasiliensis* and *S. schenckii sensu stricto* can be transmitted directly in the yeast phase through traumatic inoculation via an infected cat or via contact with mucosal tissue via respiratory droplets containing the fungus [3,4].

The molecular diagnostic approach typically involves PCR followed by Sanger sequencing, targeting specific genomic segments in fungi, which enables species identification. A frequently utilized genomic region for fungal species identification is the Internal transcribed spacer (ITS) region situated within the ribosomal RNA gene (rRNA gene), which was reported to be sufficient to identify the species in clinical clade of *Sporothrix* [2,5]. However, alternative regions are frequently used of which the calmodulin gene (exons 3–5) allows most accurate and precise phylogenetic classification and has been proposed as the gold standard for identification of *Sporothrix* species [2,6]. Recently, there were some studies indicating that the *Sporothrix* species found in Thailand, both in the capital city and other regions including the South, were mostly identified as *S. schenckii sensu stricto* rather than other species based on calmodulin (exon 3–5) sequencing [7–9].

Oxford Nanopore Technology (ONT), a third-generation sequencing method, can sequence large DNA barcode [10,11] supporting subsequent analyses such as phylogenetic analyses or microbiome studies, although, the reported high sequencing error rate may result in inaccurate species identification [12]. The objective of this study is to evaluate the performance of ONT sequencing for molecular identification and phylogenetic analysis of *Sporothrix* isolates obtained from both cats and humans in Bangkok, Thailand. The study also evaluates clustering and error-correction methods to improve the accuracy of species identification and phylogenetic classification from ONT data. Furthermore, the study includes antifungal susceptibility testing to evaluate the *in vitro* response of the clinical isolates to commonly used antifungal agents. This study hypothesizes that the proper processing and correction of high-throughput sequencing data generated by ONT can produce highly accurate representative sequences, yielding phylogenetic analyses comparable to those obtained from Sanger sequencing which is the current gold standard.

## Materials and methods

### Ethics statement

The study was conducted with approval of the Internal Review Board (IRB) of the Faculty of Medicine, Chulalongkorn University (IRB COA number 0677/66). This study was exempt from obtaining informed consent as it utilized fully anonymized data, with no patient-related information disclosed or involved.

### Isolation of clinical clade’s *Sporothrix*

A total of 26 clinical isolates were collected from confirmed clinical cases of sporotrichosis over a one-year period, between August 2022 and July 2023, from two healthcare institutes: 18 feline cases from the Faculty of Veterinary Science, Chulalongkorn University, and 8 human cases from King Chulalongkorn Memorial Hospital (KCMH). These isolates were subsequently subjected to both fungal culture on Potato dextrose agar (PDA) (Himedia, India) at 35°C for 7 days and downstream molecular assays [7,13].

### DNA extraction and PCR amplification of calmodulin and ITS region

The genomic DNA (gDNA) was extracted starting from a single colony of *Sporothrix* isolates using an suspension in the in-house developed lysis buffer (0.2 M NaCl, 0.02 M EDTA, 0.04 M Tris, 0.5% w/v SDS, 0.5% v/v β-mercaptoethanol and 100 µg/mL proteinase k) at 65°C for 3 hours and bead-beating for 3 minutes before proceeding to Maxwell RSC Whole Blood DNA Kit (Promega, Madison, USA). The extracted gDNA was then eluted with 40 µL nuclease-free molecular-graded water. The DNA quantification was determined on the NanoDrop (Thermo Fisher Scientific, Waltham, USA). We portioned the extracted gDNA into two equal aliquots before proceeding to the amplification. Each aliquot was used for amplifying the calmodulin gene (exon 3–5) and the ITS region, separately. The calmodulin gene (exon 3–5) was amplified with primers CL1 (5-GAR TWC AAG GAG GCC TTC TC-3′) and CL2A (5′-TTT TTG CAT CAT GAG TTG GAC-3′) [14]. The full ITS region of the rRNA gene of all isolates was amplified with the primers ITS1 (5′ TCCGTAGGTGAACCTGCGG 3′) and ITS4 (5′ TCCTCCGCTTATTGATATGC 3′) as previously described [15]. The polymerase chain reaction (PCR) of calmodulin was set as follows: initial denaturation at 95°C for 5 minutes; 25 cycles of 95°C for 1 minute, 55°C for 25 seconds, and 72°C for 30 seconds; final extension at 72°C for 5 minutes. The PCR of ITS was identical except for annealing at 56°C.

### Library preparation and sequencing

The amplicons were purified using the QIAquick PCR Purification Kit (QIAGEN, Maryland, USA), following the manufacturer’s prescribed protocol. The purified PCR products were quantified using the Promega Quantus Fluorometer (catalog No. E6150, Promega, Madison, USA). Subsequently, they were converted into femtomolar concentrations using NEBioCalculator v1.14 and adjusted to a concentration of 100 femtomoles. Next, DNA libraries were prepared using the Ligation Sequencing Kit (ONT, Didcot, UK), with adaptations made to the manufacturer’s guidelines. Initially, the DNA amplicons were modified using the NEBNext Ultra II End Repair/dA-tailing Module reagents (New England Biolabs, Ipswich, UK). Subsequently, the DNA was cleaned using an equal volume of Agencourt AMPure XP beads (Beckman Coulter, Indiana, USA). Next, barcodes from the Native Barcoding Expansion 1–12 (ONT, Didcot, UK) were multiplexed and attached to each isolate’s DNA sequence using Blunt/TA Ligase Master Mix (New England Biolabs, Ipswich, UK). Equimolar amounts of each barcode (approximately 12 femtomoles each) were pooled together. Adapter mix (AMII) from the Native Barcoding Expansion 1–12 (catalog No. EXP-NBD104, ONT, Didcot, UK) was attached to the DNA sample from the previous step. Subsequently, the samples were cleaned again using 0.5x volume of Agencourt AMPure XP beads, according to the manufacturer’s instructions. The libraries were mixed with Sequencing Buffer and Loading Beads in a final volume of 75 μL (100 femtomoles). The final mixture was added dropwise to the SpotON sample port. The sequencing process was conducted using the MinION flow cell R9.4.1 (ONT, Didcot, UK), following the manufacturer’s protocol with assistance from the MinKNOW graphic user interface (version 21.11.8). Base-calling, adapter trimming, and demultiplexing were carried out using Guppy (version 5.0.16, ONT) with the super accuracy mode, and a minimum Phred quality score (q-score) threshold of 15 was applied to generate the raw Fastq files. The Fastq files of both regions (ITS and calmodulin) from all isolates were filtered by Phred q score of 20 using Filtlong (ver. 0.2.1, https://github.com/rrwick/Filtlong). The Fastq files were converted to Fasta format and 10,000 reads of each isolate were subsampling using SeqKit [16]. In instances where the read count was below 10,000, all available reads were also included in the analysis. The sequence data of both regions was submitted to NCBI under the BioProject accession PRJNA1146718.

Sanger sequencing of the calmodulin gene (exon 3–5) was also conducted using an ABI 3730 genetic analyzer (Applied Biosystems, Foster City, CA, USA) following the manufacturer’s protocol. The resulting sequences have been deposited in the NCBI GenBank database under accession numbers PV520085–PV520110. Phylogenetic analysis was performed using the Maximum Likelihood (ML) method with 1000 bootstrap replicates with MEGA X.

### Read clustering, selection representative read per cluster and BLAST

Reads of each isolate were grouped with two different clustering algorithms. The first one is clustering based on sequence homology using VSEARCH [17]. The distance boundaries between two clusters were set to 1% mismatch to be excluded to another cluster. The chimeric reads and singletons were filtered during the clustering steps. After that, the representative read of each cluster (the centroid sequence as described previously [17]) was used for species classification using BLAST+ [18] with manually downloaded sequences from the NCBI database of both calmodulin (using search keyword Sporothrix [Organism] AND “Calmodulin”) and ITS (using keyword Sporothrix [Organism] AND “Internal Transcribed Spacer”) of *Sporothrix* species. The pipeline command of analysis with VSEARCH and species classification was given (S1 Data).

The second clustering method used the modified version of the NanoCLUST [19] pipeline as described elsewhere [10]. The subsampling reads were clustered and corrected with the pipeline that implemented the clustering method of Hierarchical Density-Based Spatial Clustering of Applications with Noise (HDBSCAN) [20] and Uniform Manifold Approximation and Projection (UMAP) [21] which clustered the data from the density of similar data (here the k-mer profile of the reads). The representative sequence of each cluster (selected as described previously [10]) was used for species classification using BLAST+ [18]. The identification results and number of Operational Taxonomic Unit (OTU) of all isolates and clustering method were visualized with bar plot or scatter plot generated with ggplot2 [22] and tidyverse packages [23] using RStudio.

### Representative sequence selection per isolate and phylogenetic analysis

A representative sequence for each isolate was used for species identification. First, for the clustering method using VSEARCH, the representative sequence for each isolate was selected from the cluster with the largest number of supported reads (the most abundant cluster). Regarding the UMAP-HDBSCAN pipeline, the representative sequence was chosen among the representative sequence of all the pass clusters by comparing the Average Nucleotide Identity (ANI) scores between all pair of sequences using FastANI [24]. The sequence with the highest average ANI score was chosen as the final consensus sequence of that isolate. The representative sequence of each isolate was then used for species identification via BLAST+ [18].

To construct the phylogenetic trees, the representative sequence of each isolate was used. The analysis was performed using the maximum likelihood (ML) method with 1,000 bootstrap replicates, and the Kimura-2-parameter distance model with a gamma distribution (K2P+G) [25] was selected. Phylogenetic tree construction was carried out using MEGA X [26]. In addition to the representative calmodulin sequences obtained in this study, calmodulin sequences from previously reported *Sporothrix* strains were included for the phylogenetic analysis (S1 Table). Calmodulin sequences of two *Aspergillus* strains and three strains from other genera in the Ophiostomataceae family were included as outgroups. The evolutionary distance model used for phylogenetic tree construction was selected based on the model with the lowest Bayesian Information Criterion (BIC) and Akaike Information Criterion (AIC), incorporating a correction for small sample sizes, as previously described [27]. Ancestral state reconstruction was subsequently performed using the maximum likelihood method with the same distance model employed in earlier genotyping studies of clinical *Sporothrix* isolates from Thailand [7–9]. In addition, Tajima’s D neutrality test was conducted to assess the evolutionary dynamics of the studied sequences [28].

### Antifungal susceptibility testing

The antifungal susceptibility testing was performed according to the Clinical and Laboratory Standards Institute (CLSI M27-A2) using Sensititre YeastOne YO10 (Thermo Scientific, USA) [7,13]. Prior to antifungal susceptibility testing, isolates were subcultured on PDA and incubated at 37°C for 7 days. Our study evaluated minimum inhibitory concentrations (MIC) of 5 antifungal drugs including amphotericin B (AMB), fluconazole (FLC), itraconazole (ITC), posaconazole (POS), and voriconazole (VRC). The inoculum was prepared by suspending fungal cells to a final concentration of 10^3^ colony-forming units (CFU/mL). The inoculated microplates were incubated at 35°C and read after 72 hours by ViZION (observation of a colorimetric indicator: alamarBlue and microscopic examination). The MIC inhibiting 50% (MIC_50_) and 90% (MIC_90_) of isolates was calculated along with the MIC range and the geometric means of the MIC values. Epidemiological cut-off values (ECVs) for AMB, ITC, POS and VRC were implemented according to Espinel-Ingroff [29].

## Results

### Cluster analysis of calmodulin and ITS reads of *Sporothrix* isolates

**Fig 1:**
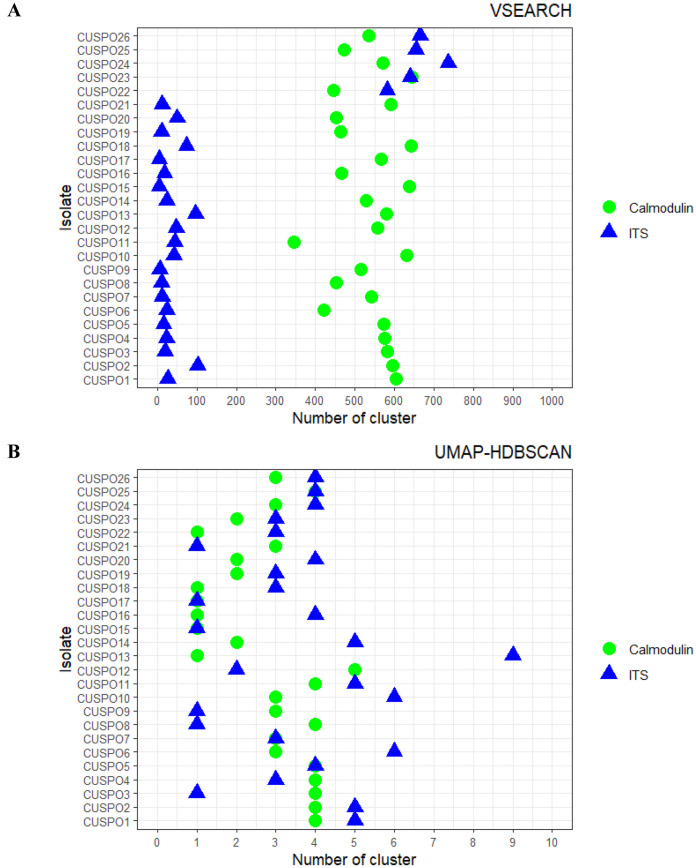
The number of clusters via two different clustering methods. Reads of each isolate were grouped with **(A)** VSEARCH and **(B)** UMAP-HDBSCAN and calmodulin and ITS sequencing are shown in different shape and color.

In this study we included 18 isolates from feline sporotrichosis, mostly from lesions, legs and nostrils, and eight isolates from human sporotrichosis, mostly collected from eyes and subcutaneous lesions. To identify the species level of each *Sporothrix* isolate, the calmodulin and ITS regions were amplified and sequenced using ONT. To obtain a representative calmodulin and ITS sequence per isolate, reads of each isolate were clustered using the algorithms VSEARCH and UMAP-HDBSCAN. First, the number of clusters were projected from both algorithms were highly different. While VSEARCH projected 345–645 and 6–740 clusters for calmodulin and ITS, respectively (Fig 1A), there were only 1–5 and 1–9 clusters for calmodulin and ITS, respectively, with UMAP-HDBSCAN (Fig 1B).

**Fig 2:**
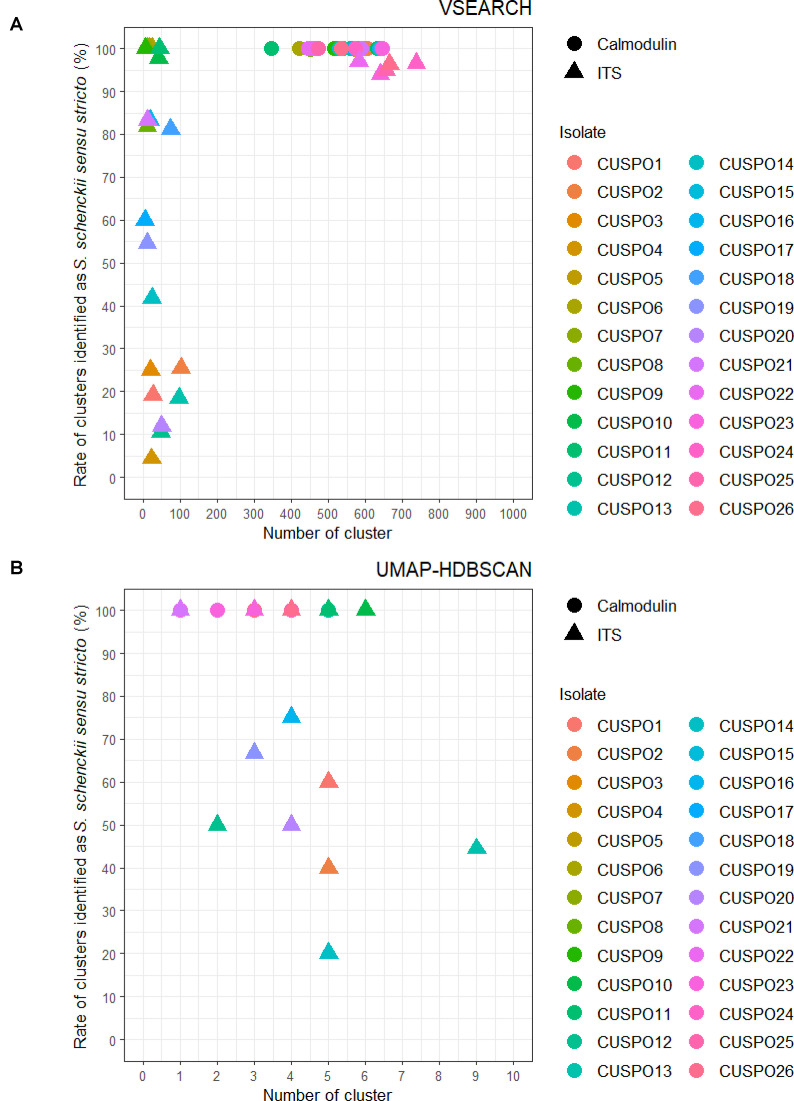
Distribution of identification pattern of clusters projected from different clustering methods. Rate of clusters identified as *S. schenckii sensu stricto* (%) using **(A)** VSEARCH or **(B)** UMAP-HDBSCAN using calmodulin or ITS sequences. Calmodulin and ITS are shown with different shapes, each isolate with a different color.

Next, the representative read of each cluster was used to identify the species level of each isolate using BLAST+ [18] on 2,340 manually downloaded calmodulin and 1,519 ITS sequences of *Sporothrix* species from the NCBI database on 1/5/2023. With the calmodulin sequences, *S. schenckii sensu stricto* was identified for all clusters using both VSEARCH (Fig 2A) and UMAP-HDBSCAN (Fig 2B). Using ITS sequences, some isolates were also consistently identified as *S. schenckii sensu stricto* across all clusters, regardless of the clustering method. However, for other isolates, additional clusters were identified as different *Sporothrix* species alongside those identified as *S. schenckii sensu stricto* (Fig 2). Specifically, with VSEARCH and UMAP-HDBSCAN there were 14 and 8 isolates, respectively, in which some of the clusters were identified as *Sporothrix* species other than *S. schenckii sensu stricto*. Notably, with VSEARCH and UMAP-HDBSCAN there were respectively 8 and 3 isolates in which less than 50% of the total clusters was classified as *S. schenckii sensu stricto* (Fig 2).

### Identification with representative sequence of calmodulin and ITS of *Sporothrix* isolates

**Fig 3:**
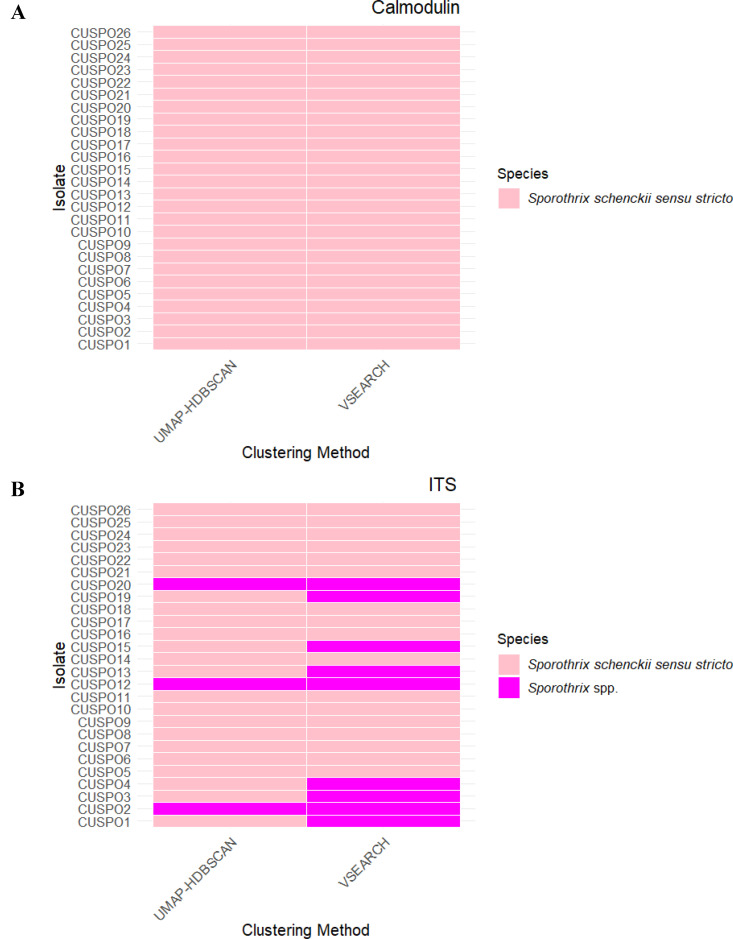
Identification of selected representative sequence from each method with calmodulin and ITS. Isolates with representative sequences from calmodulin(A) and ITS (B) that were identified as *S. schenckii sensu stricto* were marked in pink and isolates only identified to the *Sporothrix* genus level were marked in magenta.

After clustering the calmodulin and ITS sequences with two different algorithms, a representative sequence of each isolate was selected. For VSEARCH, the representative sequence was selected from the cluster with the largest number of supported reads, while for UMAP-HDBSCAN the consensus sequence of all the pass clusters with the highest average ANI score was selected. BLASTing of all representative calmodulin sequences from either VSEARCH or UMAP-HDBSCAN resulted in the identification of all isolates as *S. schenckii sensu stricto* with a percent identity range of 99–100% (Fig 3A and S2 Table). For the ITS region most isolates were also identified as *S. schenckii sensu stricto* with a percent identity range 80.44%-100% (S2 Table) except for isolate CUSPO2, CUPO12, and CUSPO20 by UMAP-HDBSCAN clustering and isolate CUSPO1, CUSPO2, CUSPO3, CUSPO4, CUSPO12, CUSPO13, CUSPO15, CUSPO19 and CUSPO20 using VSEARCH clustering, which were identified as other *Sporothrix* species (Fig 3B).

### Phylogenetic analysis of sequence clustered from UMAP-HDBSCAN pipeline

**Fig 4:**
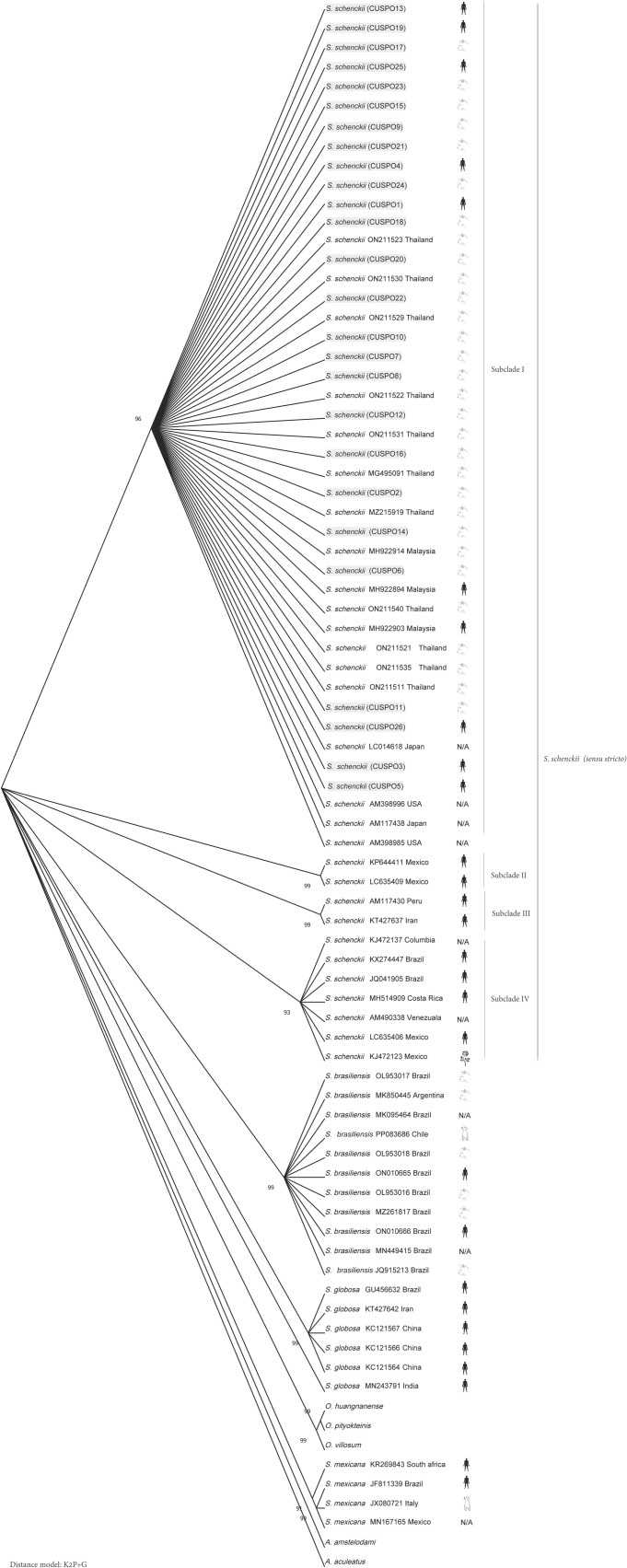
Bootstrap consensus tree of calmodulin sequences of *Sporothrix* isolates. The tree was generated using the maximum likelihood (ML) method with 1,000 bootstrap replicates with K2P+G distance model, representative sequence from polished ONT reads. The tested isolates in this study were highlighted in light grey in the format of species (isolate name) and with picture depicted the host species. The isolates which have ambiguous host information were labeled “N/A” (not available). Host graphics used in the figure were obtained as followed: cat (https://openclipart.org/detail/308557/cat-icon-grey), dog (https://openclipart.org/detail/8499/dog-simple-drawing), human (https://openclipart.org/detail/182185/man-shape) and rose (https://openclipart.org/detail/221383/rose-silhouette) and were released under the Creative Commons Zero 1.0 Public Domain License (CC0 1.0).

Having identified all isolates as *S. schenckii sensu stricto* with calmodulin sequencing, we aimed to determine the relatedness between the isolates in this study and *S. schenckii sensu stricto* isolates previously reported. As ITS sequencing did not identify all isolates to the species level, calmodulin sequences were used to construct a phylogenetic tree. All *S. schenckii sensu stricto* isolates from this study were used, with the addition of isolates from other studies including *S. schenckii sensu stricto*, different *Sporothrix* species, and as outgroups two *Aspergillus* strains and three strains from other genera in the Ophiostomataceae family (S1 Table). All our isolates grouped with isolates previously reported as *S. schenckii sensu stricto*, which were divided in four distinct subclades (Fig 4). Subclade I included isolates of both human and cat from Thailand (Southern part and capital city), Malaysia (human and cat), Japan (human and cat), USA (human only) and also all isolates from this study. Subclade II was solely from Mexico, while subclade III isolates originated from clinical human cases from Iran and Peru. Lastly, subclade IV mostly consisted of isolates of clinical human cases from South America. Isolates with ambiguous host information were labeled “N/A” (not available). An ancestor estimation analysis using the maximum likelihood (ML) method was performed (S1 Fig) yielding a Tajima’s *D* neutrality test of *D* = 0 (*P*-value < 0.05).

To confirm the phylogenetic placement of the isolates sequenced using Oxford Nanopore Technologies (ONT), we also performed Sanger sequencing, considered as the gold standard method, to build a phylogenetic tree. Sequencing the calmodulin gene (exon 3–5) confirmed the ONT results that all isolates from this study clustered within the *S. schenckii sensu stricto* clade, consistent with prior classifications. Within this clade, four distinct subclades were observed (S2 Fig). The isolates which have ambiguous host information were labeled “N/A” (not available).

### Antifungal susceptibility profile of *Sporothrix* isolates

Antifungal susceptibility testing using amphotericin B and four azole drugs was performed on all isolates (Table 1) and the MIC range, MIC_50_ and MIC_90_ and geometric mean of the MIC value were determined (Table 1)_._ Most isolates were found to show highly elevated MICs to fluconazole (FLC) with a MIC range of 32 to >256 µg/mL. The MIC_50_, MIC_90_, and geometric mean were 256 µg/mL, > 256 µg/mL and 134.50 µg/mL, respectively. For itraconazole (ITC), the drug of choice for sporotrichosis, a wide MIC range of 0.06 to 8 µg/mL was identified. The MIC_50_, MIC_90_, and geometric mean were 0.5 µg/mL, 8 µg/mL and 1.05 µg/mL, respectively. Amphotericin B (AMB) demonstrated a MIC range of 1–4 µg/mL. The MIC_50_, MIC_90_, and geometric mean were 4 µg/mL, 4 µg/mL and 2.83 µg/mL, respectively. Posaconazole (POS) showed a MIC range of 0.06 to 1 µg/mL. The MIC_50_, MIC_90_, and geometric mean were 0.5 µg/mL, 1 µg/mL and 0.53 µg/mL, respectively. Finally, Voriconazole (VRC) had a MIC range of 0.25 to 2 µg/mL. The MIC_50_, MIC_90_, and geometric mean were 1 µg/mL, 2 µg/mL and 0.64 µg/mL, respectively. Comparing MIC levels with the tentative ECV proposed by Espinel-Ingroff et al, there were eight isolates with elevated MIC levels for ITC, while none of the isolates demonstrated MIC levels above the ECV for AMB, POS and VRC (Table 1) [29].

**Table 1:** Metadata and antifungal susceptibility profiles of isolates.

Isolate	Host	Amphoterricin B (µg/mL)	Fluconazole (µg/mL)	Itraconazole (µg/mL)	Posaconazole (µg/mL)	Voriconazole (µg/mL)
CUSPO1	Human	1	256	0.25	0.25	1
CUSPO2	Cat	4	>256	0.5	1	2
CUSPO3	Human	4	>256	0.5	0.5	2
CUSPO4	Human	4	>256	0.5	1	2
CUSPO5	Human	4	128	0.5	0.5	1
CUSPO6	Cat	4	>256	8	1	0.5
CUSPO7	Cat	2	>256	8	0.5	0.25
CUSPO8	Cat	2	>256	8	0.5	0.25
CUSPO9	Cat	2	32	0.06	0.06	0.25
CUSPO10	Cat	4	128	1	0.5	0.25
CUSPO11	Cat	1	128	0.25	0.5	1
CUSPO12	Cat	2	256	0.25	0.25	1
CUSPO13	Human	2	128	0.25	0.25	0.5
CUSPO14	Cat	4	>256	1	1	0.25
CUSPO15	Cat	1	128	0.25	0.25	0.5
CUSPO16	Cat	2	256	0.25	0.25	1
CUSPO17	Cat	4	32	0.5	0.5	0.25
CUSPO18	Cat	4	256	0.5	0.5	1
CUSPO19	Human	4	>256	8	0.5	1
CUSPO20	Cat	4	>256	1	1	1
CUSPO21	Cat	4	>256	8	1	1
CUSPO22	Cat	4	128	8	1	0.5
CUSPO23	Cat	4	128	8	1	0.5
CUSPO24	Cat	2	256	1	0.5	0.25
CUSPO25	Human	4	>256	8	1	1
CUSPO26	Human	4	>256	1	1	0.5
MIC_50_ (µg/mL)		4	256	0.5	0.5	1
MIC_90_ (µg/mL)		4	>256	8	1	2
MIC range (µg/mL)		1-4	32- > 256	0.06-8	0.06-1	0.25-2
Geometric mean		2.83	134.50	1.05	0.53	0.64

Details about the isolates included in this study, including their identification number, host species and susceptibility profiles. The susceptibility profiles are presented as Minimal Inhibitory Concentration (MIC) values for the antifungal drugs amphotericin B, fluconazole, itraconazole, posaconazole, and voriconazole. The MIC values are also presented as MIC_50_, MIC_90_, MIC range, and geometric mean of MIC values.

## Discussion

This study determined the species and genetic relatedness of human and feline *Sporothrix* isolates from Thailand and analyzed their antifungal susceptibility —an important clinical feature, as *Sporothrix* species are showing reduced responsiveness to treatment [29]. The first aim of the study was to determine the species and the genetic relatedness of the *Sporothrix* isolates by sequencing calmodulin and ITS genes using ONT. Prior studies highlighted significant intraspecific variability in ITS among *S. schenckii* complex isolates [30,31], while the calmodulin region yielded a more discriminative phylogenetic tree for the clinical clade *Sporothrix*. Furthermore, ONT sequencing faced high error rates [32]. With these challenges in mind, this study applied two bioinformatic methods analyze calmodulin and ITS sequences generated by ONT and to determine whether we could reliably perform species identification and phylogenetic analysis. With the first method, based on VSEARCH clustering, a homology threshold of approximately 99% was applied, thereby generating a high number of clusters, which might be due to nanopore’s inherent sequence error rate [33]. With calmodulin 345–645 clusters were found per isolate, while for ITS most isolates showed less than 50 clusters. In our study the high number of clusters did not seem to hinder an accurate analysis as calmodulin sequencing lead to species identification of all isolates, while ITS sequencing with VSEARCH clustering led to more pleomorphic clusters, including non-*S. schenckii* sequences, despite the smaller number of clusters. Nonetheless, previous research suggested that the optimal similarity threshold for ITS sequencing with ONT should be considerably lower than 99%, in order to group a larger number of reads into single clusters [11]. Contrarily, too much reduction of the similarity threshold may inadvertently include sequences with greater variability [11], which could lead to a lower resolution especially when analyzing shorter sequences, potentially complicating taxonomic assignments. Furthermore, the study demonstrated that the resolution of clustering based on the full ITS region was lower compared to that from sequences of the calmodulin gene [11]. As such, the determination of an ideal similarity threshold remains an open challenge, requiring further optimization to enhance both clustering accuracy and taxonomic resolution.

The second method involves k-mer frequency-based UMAP dimensionality reduction followed by HDBSCAN clustering as described previously [10]. This pipeline organizes highly heterogeneous sequences by reducing the data to k-mer profiles and comparing these profiles instead of the entire sequences [34]. The clustering was based on regions with high data density rather than attempting to cluster all sequences, while reads with high error rates were excluded from clustering [35]. This approach effectively reduced the number of clusters for both calmodulin gene and ITS sequences from several hundreds to 9 clusters at most. While UMAP-HDBSCAN successfully separated the clusters, it also showed limitations for high-variation regions like ITS, where clusters sometimes failed to align with the intended species due to biological variation or noise from the sequencing technology itself. Comparing both clustering methods in regard to the final consensus sequence, we found that both methods lead to 100% species identification for calmodulin, while for the ITS region, UMAP-HDBSCAN analysis resulted in 3/26 (10.5%) of isolates in other species than *S. schenckii sensu stricto*, which was 9/26 (34.6%) with VSEARCH. Thus, calmodulin gene seems more appropriate for the identification of *S. schenckii* complex species with ONT.

This study also investigated the genetic relatedness using the calmodulin region sequenced with ONT. All isolates in this study, identified as *S. schenckii sensu stricto*, were found to locate in the same *S. schenckii* subclade I. This subclade includes previously reported isolates from Thailand [8,9], Malaysia [36], Japan [37] and the USA [38]. However, some isolates from the USA and Japan presented a limitation due to incomplete metadata, particularly regarding their host origin. Two sequences from the USA were confirmed as clinical isolates. However, the specific host was not reported [38]. These sequences nonetheless contribute to illustrating the broader global distribution of this subclade across multiple continents. In the case of the Japanese isolates, both strains also lack complete metadata. One of the strains was obtained from the Biological Resource Center in Chiba, Japan, but its original source and host information were untraceable [37]. Similarly, the other Japanese strain was directly submitted to NCBI without accompanying data on its geographic or host origin (GenBank accession LC014618). This lack of detailed metadata poses a challenge in interpreting the epidemiological significance of the Japanese isolates. Therefore, while these sequences support the phylogenetic clustering within subclade I, the absence of verified host and origin information limits the ability to draw definitive conclusions about their role in zoonotic transmission dynamics. Notably, with the exception of the USA and Japan isolates, all other sequences within this subclade were derived from either feline or human hosts. This pattern suggests the presence of a geographically associated genotype in Southeast Asia that may be involved in zoonotic transmission between cats and humans. Such a transmission route is reminiscent of the well-established zoonotic behavior of *S. brasiliensis*, a closely related species known to cause outbreaks of sporotrichosis through cat-to-human transmission [39]. These findings raise the possibility that certain *S. schenckii* strains circulating in Southeast Asia may possess a similar zoonotic potential. Until ten years ago cat-transmitted sporotrichosis via *S. schenckii sensu stricto* was not reported, but that changed with the Malaysian outbreak in 2014–2015. At the moment it is not clear whether the following reports of cat-transmitted sporotrichosis via *S. schenckii sensu stricto* in Thailand were due to the transmission of the Malaysian strain or whether there are multiple independent emergences of genetically diverse *S. schenckii sensu stricto* strains in these countries, all capable of zoonotic transmission. Although the genetic variation between *S. schenckii sensu stricto* isolates is in general quite high [40] and multiple independent emergences from the environment into cats have also been reported for *S. brasiliensis* [41], WGS analysis is required to answer this question, as all *S. schenckii sensu stricto* isolates capable of zoonotic transmission group in the same subclade with calmodulin sequencing.

Importantly, the phylogenetic tree constructed from representative consensus sequences derived from ONT showed a high degree of concordance with the tree generated from Sanger sequencing. This concordance confirms the reliability and accuracy of ONT-based consensus sequences for phylogenetic analysis of *S. schenckii sensu stricto*. The results demonstrate that, when processed through an optimized pipeline, ONT sequencing can produce high-resolution consensus sequences that are suitable for sensitive applications such as phylogenetic classification at clade- and subclade-level. This finding is particularly significant given ONT’s nature of having higher raw read error rates.

The ability of ONT sequencing to accurately resolve evolutionary relationships within *S. schenckii* highlights its value for molecular epidemiology. Furthermore, our study provides evidence that ONT can be confidently used for phylogenetic analysis. By validating ONT’s utility in generating phylogenetically informative data, this work contributes to the expansion of practical applications of third-generation sequencing technologies in mycological research and public health. The clustering algorithm and correction of multiple read clusters with ONT sequencing will also be beneficial in the future utilities like microbiome and mycobiome analysis or species identification and phylogenetic analysis in non-sterile clinical samples.

This study conducted the *in vitro* antifungal susceptibility testing including itraconazole, the drug of choice to treat both feline and human sporotrichosis. It was found that 8/26 (31%) isolates exhibited elevated itraconazole MICs (>2 µg/mL) according to the previously published tentative ECV [29]. Furthermore, the antifungal susceptibility of other feline and human *S. schenckii* isolates from the region of Southeast Asia (including Malaysia, and Thailand) that genetically allocated to the same subclade as the isolates in this study, was previously analyzed [7–9,36]. The isolates from Malaysia, collected during 2014–2015, showed relatively low MICs to itraconazole with only 2/40 (5%) isolates above the tentative ECV [7]. From the Southern part of Thailand, collected during 2018–2021, 21/38 (55%) isolates displayed elevated MICs to itraconazole, with values of >2 µg/mL [8]. Recently in 2023, another veterinary center located in Bangkok found elevated itraconazole MICs of >4 µg/mL for 4/26 (15%) isolates [9]. As we also found elevated itraconazole MICs in 31% of isolates that were collected in 2022–2023, there seems to be an increase in number of isolates with elevated itraconazole MICs in the last decade [8,9,36,37].

Other drugs including fluconazole also demonstrated relatively high MICs. It is postulated that the elevated MICs may be linked to the genomic adaptability of *S. schenckii sensu stricto*, characterized by variability in both chromosome size and count [42,43]. The observed genetic plasticity of *Sporothrix* spp. is thought to influence their *in vitro* antifungal susceptibility profiles. However, despite numerous studies evaluating the *in vitro* activity of antifungal agents against *Sporothrix* spp., these results do not always correlate with clinical efficacy *in vivo* [44,45]. As a result, these antifungal agents are still used as second-line treatments, often in combination with other drugs. A combination of different antifungal agents might yield a more favorable therapeutic response, particularly considering that some of these drugs are utilized as second-line therapies, often in conjunction with SSKI or terbinafine [46]. However, terbinafine is not commonly available by hospitals within Thailand.

Although the Sensititre YeastOne system has been widely used for antifungal susceptibility testing of various fungal pathogens due to its convenience and standardized format, its application for dimorphic fungi such as *S. schenckii* remains controversial. *Sporothrix* spp. can exhibit different antifungal susceptibility profiles depending on whether the yeast or mold phase is tested, and this dimorphism may influence MIC results [30,31]. The CLSI M38-A2 broth microdilution method would here be the preferred reference standard for antifungal susceptibility testing of filamentous fungi [29], providing greater consistency and comparability across studies. We recognize that MIC values obtained using commercial systems may differ from those obtained by CLSI reference methods of filamentous fungi. As such, results should be interpreted cautiously.

Overall, this study highlights the growing concern of elevated itraconazole MICs in some isolates of *Sporothrix schenckii sensu stricto* isolates from Thailand, emphasizing the need for ongoing surveillance of antifungal susceptibility profiles. Comparative assessments of clustering methods demonstrated that k-mer frequency-based UMAP-HDBSCAN clustering reduced technical errors and improved representative sequence selection, particularly for the ITS region that it chose the representative sequence more correctly. While further optimization of clustering thresholds is necessary, this approach shows promise for analyzing complex, high-variation datasets generated through high-throughput sequencing. In this context, long-read sequencing technologies, such as ONT, offer distinct advantages by enabling full-length sequencing of barcode regions like calmodulin (700–800 bp.). Our findings suggest that Nanopore-generated sequences can achieve taxonomic resolutions comparable to gold-standard methods such as the Sanger sequencing, supporting its potential utility for more accurate identification and phylogenetic analysis of *Sporothrix* species and other species of pathogenic or industrial fungi. These advancements contribute to improve diagnostic accuracy and a better understanding of the sporotrichosis epidemiology.

## Supporting information

S1 Fig Phylogenetic analysis for estimation of ancestor using calmodulin region. The ancestor was estimated using Maximal Likelihood (ML) model and Tajima’s *D* neutrality.
(TIF)

S2 Fig Phylogenetic analysis of *Sporothrix* isolates with calmodulin region using Sanger sequencing. The tree was generated using the maximum likelihood (ML) method with 1,000 bootstrap replicates with K2P+G distance model. The tested isolates in this study were highlighted in light grey in the format of species (isolate name) and with picture depicted the host species. Host graphics used in the figure were obtained from https://openclipart.org/, and are released under the Creative Commons Zero 1.0 Public Domain License (CC0 1.0).
(TIF)

S1 Table Profile of isolates included for phylogenetic analysis via calmodulin. (XLSX)

S2 Table BLAST+ results of representative calmodulin and ITS sequences from UMAP-HDBSCAN. (XLSX)

S1 Data The command lines for each of the programs used in the study. (DOCX)
