# Supplementary figures and images for: Targeted long-read sequencing analysis and antifungal susceptibility profiles of Sporothrix schenckii isolates from Thailand
Source: PLoS Negl Trop Dis. 2025 Jun 30;19(6):e0013253. doi: 10.1371/journal.pntd.0013253 (PMC12233950; doi:10.1371/journal.pntd.0013253)

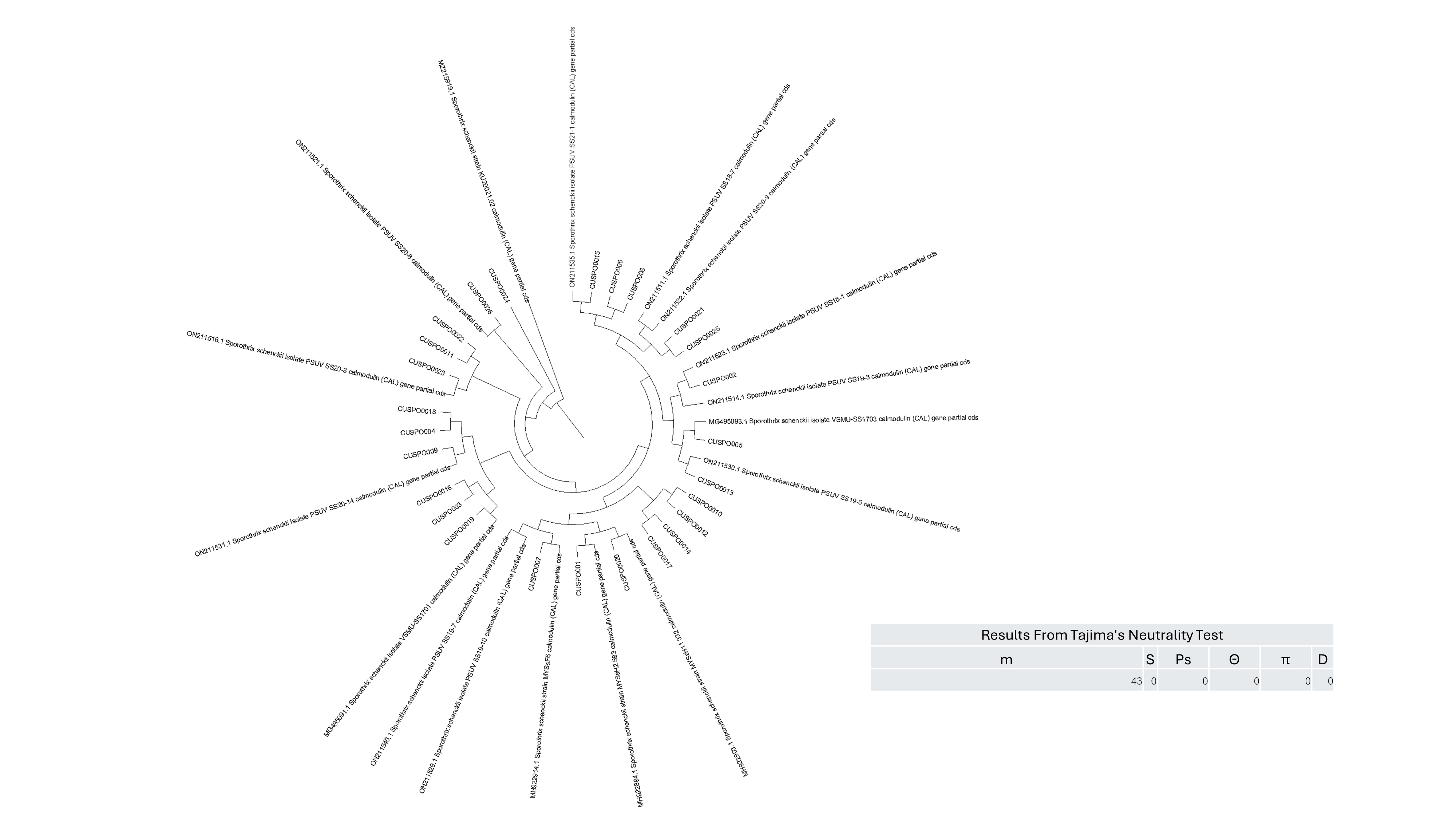

Supplement: S1 Fig — The ancestor was estimated using Maximal Likelihood (ML) model and Tajima’s D neutrality. (TIF) [file pntd.0013253.s001.tif]
